# Supplementary material for: Genetic perturbation of cellular homeostasis regulates integrated stress response signaling to control Drosophila hematopoiesis
Source: Biol Open. 2025 Jul 8;14(7):bio062046. doi: 10.1242/bio.062046 (PMC12536935; doi:10.1242/bio.062046)
Supplement: Supplementary information [file biolopen-14-062046-s1.pdf]

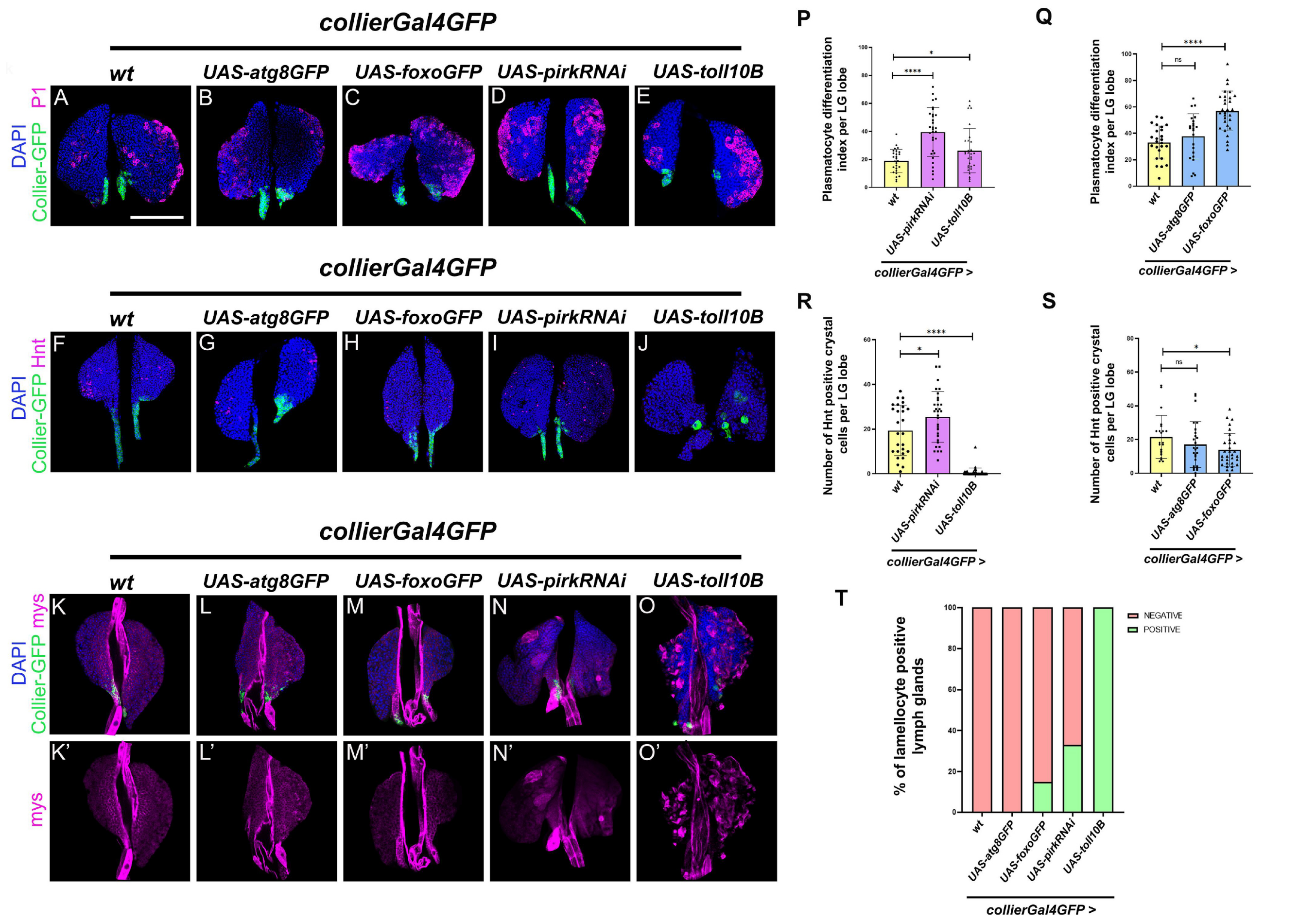

Fig. S1. PSC niche-specific modulation of cellular homeostasis affects LG hematopoiesis.

Plasmatocyte differentiation marked by P1 (magenta) or crystal cell differentiation marked by Hindsight (Hnt) (magenta) or lamellocyte differentiation marked by β-integrin-specific Myospheroid (Mys) (magenta) upon PSC niche-specific (using *collierGal4*) expression of *UAS-atg8GFP* (Fig. S1B, G, L), *UAS-foxoGFP* (Fig. S1C, H, M), *UAS-pirkRNAi* (Fig. S1D, I, N) or *UAS-toll10B* (Fig. S1E, J, O) as compared to *wildtype* control (Fig. S1A, F, K). GFP expression (green) is driven by *collierGal4* (Fig. S1A-O). Nuclei are stained with DAPI (Blue). Graphical representation of plasmatocyte differentiation index or number of crystal cells or percentage of lamellocyte positive lymph glands upon *collierGal4* mediated expression of *UAS-pirkRNAi* or *UAS-toll10B* (Fig. S1P, R, T) or *UAS-atg8GFP* or *UAS-foxoGFP* (Fog. S1Q, S, T) as compared to *wildtype* control. For Plasmatocyte differentiation: *collierGal4 x UAS-pirkRNAi* (N = 16 , n = 31) and *collierGal4 x UAS-toll10B* (N = 16, n = 31) as compared to *collierGal4 x wt* (N = 14, n = 27), *collierGal4 x UAS-atg8GFP* (N = 12, n = 23), *collierGal4 x UAS-foxoGFP* (N = 15 , n = 30) as compared to *collierGal4 x wt* (N = 13 , n = 25). For crystal cell numbers: *collierGal4 x UAS-pirkRNAi* (N = 16 , n = 31) and *collierGal4 x UAS-toll10B* (N = 20, n = 40) as compared to *collierGal4 x wt* (N = 14, n = 27), *collierGal4 x UAS-atg8GFP* (N = 13, n = 26), *collierGal4 x UAS-foxoGFP* (N = 14 , n = 28) as compared to *collierGal4 x wt* (N = 11, n = 22). N denotes the number of larvae & n denotes the number of individual primary lymph gland lobes analysed per genotype. Individual data points in the graphs represent individual primary lobes of the Lymph gland. Values are mean ± SD, and asterisks denote statistically significant differences (ns denotes not significant, *p<0.05, **p<0.01, *** p<0.001, ****p<0.0001). Student's t-test with Welch's correction was performed for the statistical analysis. Scale Bar: 50 µm (A-O').

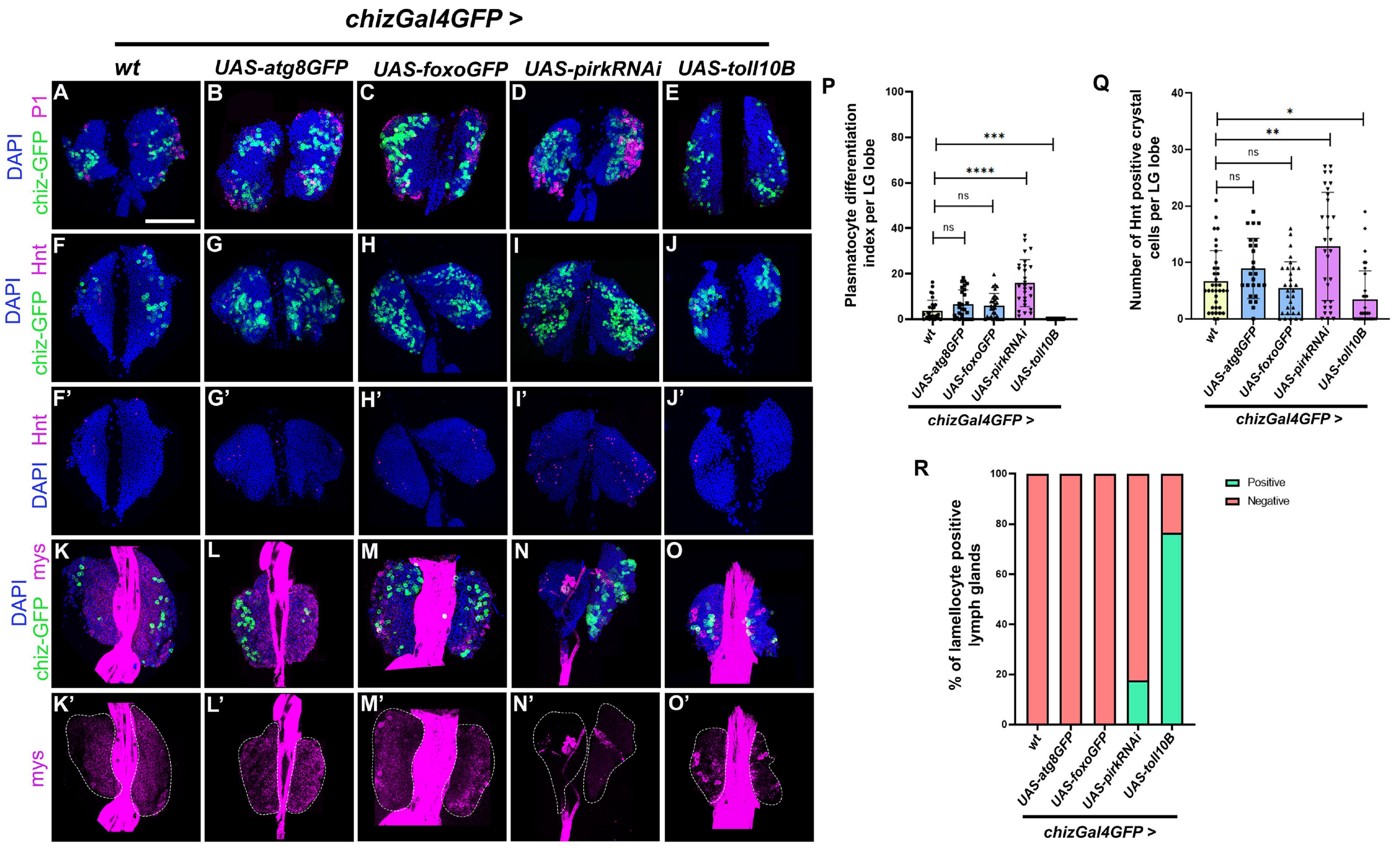

Fig. S2. Intermediate progenitor-specific modulation of cellular homeostasis alters LG hematopoiesis.

Plasmatocyte differentiation marked by P1 (magenta) or crystal cell differentiation marked by Hindsight (Hnt, magenta) or lamellocyte differentiation marked by β-integrin-specific Myospheroid (Mys, (magenta) upon intermediate progenitor-specific (using *chizGal4*) expression of *UAS-atg8GFP* (Fig. S2B, G, L), *UAS-foxoGFP* (Fig. S2C, H, M), *UAS-pirkRNAi* (Fig. S2D, I, N) or *UAS-toll10B* (Fig. S2E, J, O) as compared to *wildtype* control (Fig. S2A, F, K). GFP expression (green) is driven by *chizGal4* (Fig. S2A-O). Nuclei are stained with DAPI (Blue). Graphical representation of plasmatocyte differentiation index (Fig. S2P) or number of crystal cells (Fig. S2Q) or percentage of lamellocyte positive lymph glands (Fig. S2R) upon *chizGal4* mediated expression of *UAS-pirkRNAi, UAS-toll10B, UAS- atg8GFP* or *UAS-foxoGFP* as compared to *wildtype* control. For Plasmatocyte differentiation, *chizGal4 x UAS-pirkRNAi* (N = 14, n = 28), *chizGal4 x UAS-toll10B* (N = 15, n = 30), *chizGal4 x UAS-atg8GFP* (N = 14, n = 28), *chizGal4 x UAS-foxoGFP* (N = 15, n = 30) were analyzed as compared to *chizGal4 x wt* (N = 15, n = 30). For crystal cell numbers, *chizGal4 x UAS- pirkRNAi* (N = 16, n = 32), *chizGal4 x UAS-toll10B* (N = 16, n = 32), *chizGal4 x UAS- atg8GFP* (N = 14, n = 27), *chizGal4 x UAS-foxoGFP* (N = 15, n = 30) were analyzed as compared to *chizGal4 x wt* (N = 18, n = 35). For lamellocyte differentiation, *chizGal4 x UAS- pirkRNAi* (N = 16), *chizGal4 x UAS-toll10B* (N = 14), *chizGal4 x UAS-atg8GFP* (N = 19), *chizGal4 x UAS-foxoGFP* (N= 16) were analyzed as compared to *chizGal4 x wt* (N = 15). N denotes the number of larvae & n denotes the number of individual primary lymph gland lobes analyzed per genotype. Individual data points in the graphs represent individual primary lobes of the Lymph gland. Values are mean ± SD, and asterisks denote statistically significant differences (ns denotes not significant, *p<0.05, **p<0.01, ***p<0.001, ****p<0.0001). Student's t-test with Welch's correction was performed for the statistical analysis. Scale Bar: 50 µm (A-O').

Biology Open • Supplementary information

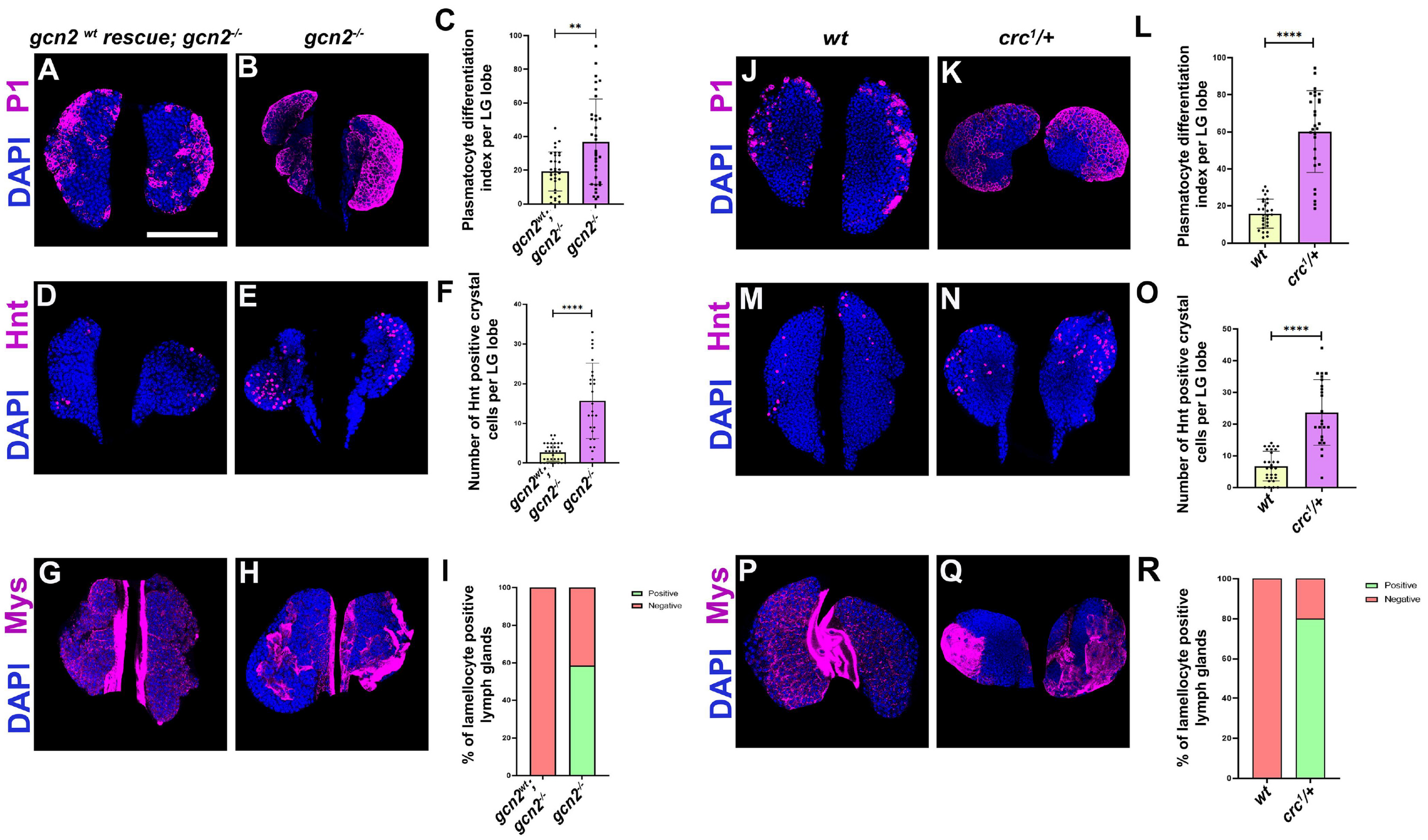

Fig. S3. ISR pathway mutants exhibit defective lymph gland homeostasis

Plasmatocyte differentiation marked by P1 (magenta, Fig. S7B, K) or crystal cell numbers marked by Hindsight (Hnt in magenta, Fig. S7E, N) or Lamellocyte differentiation marked by β-integrin specific Myospheroid (Mys in magenta, Fig. S7H, Q) in whole animal *gcn2*$^{-/-}$ homozygous null mutant or *crc*$^{1/+}$ heterozygous hypomorphic mutant LGs as compared to *gcn2*^{wt} rescue; *gcn2*$^{-/-}$ (Fig. S7A, D, G) or *wildtype* (Fig. S7J, M, P) controls respectively. Graphical representation of Plasmatocyte differentiation index (Fig. S7C, L) or Crystal cell numbers (Fig. S7F, O) or percentage of lamellocyte positive lymph glands (Fig. S7I, R) in whole animal *gcn2*$^{-/-}$ null mutant or *crc*$^{1/+}$ heterozygous mutant as compared to the respective *gcn2*^{wt} rescue; *gcn2*$^{-/-}$ (Fig. S7C, F, I) or *wildtype* (Fig. S7L, O, R) controls. *gcn2*$^{-/-}$ *and crc*$^{1/+}$ mutants for Plasmatocyte differentiation (*gcn2*^{wt} rescue; *gcn2*$^{-/-}$ - N = 17, n = 34; *gcn2*$^{-/-}$ - *N* = 17, *n = 34 and wt* – N = 15, n = 29*;* *crc*$^{1/+}$ - N = 14, n = 28) or crystal cell numbers (*gcn2*^{wt} rescue; *gcn2*$^{-/-}$ - N = 18, n = 35; *gcn2*$^{-/-}$ - *N* = 14*, n* = 27 *and wt* – *N* = 15*,* n = 29*;* *crc*$^{1/+}$ - N = 12, n = 24) or percentage of lamellocyte positive lymph glands (*gcn2*^{wt} rescue; *gcn2*$^{-/-}$ - N = 15; *gcn2*$^{-/-}$ - *N* = 15 *and wt* – *N* = 15*;* *crc*$^{1/+}$ - N = 15) were analyzed per genotype. N denotes the number of larvae & n denotes the number of individual primary lymph gland lobes analyzed per genotype. Individual data points in the graphs represent individual primary lobes of the Lymph Gland. Values are mean ± SD, and asterisks denote statistically significant differences (ns denotes not significant, **p < 0.01, *** p < 0.001, ****p < 0.0001). Student's t-test with Welch's correction was performed for the statistical analysis. Scale Bar: 50 µm (A- Q).

Biology Open • Supplementary information

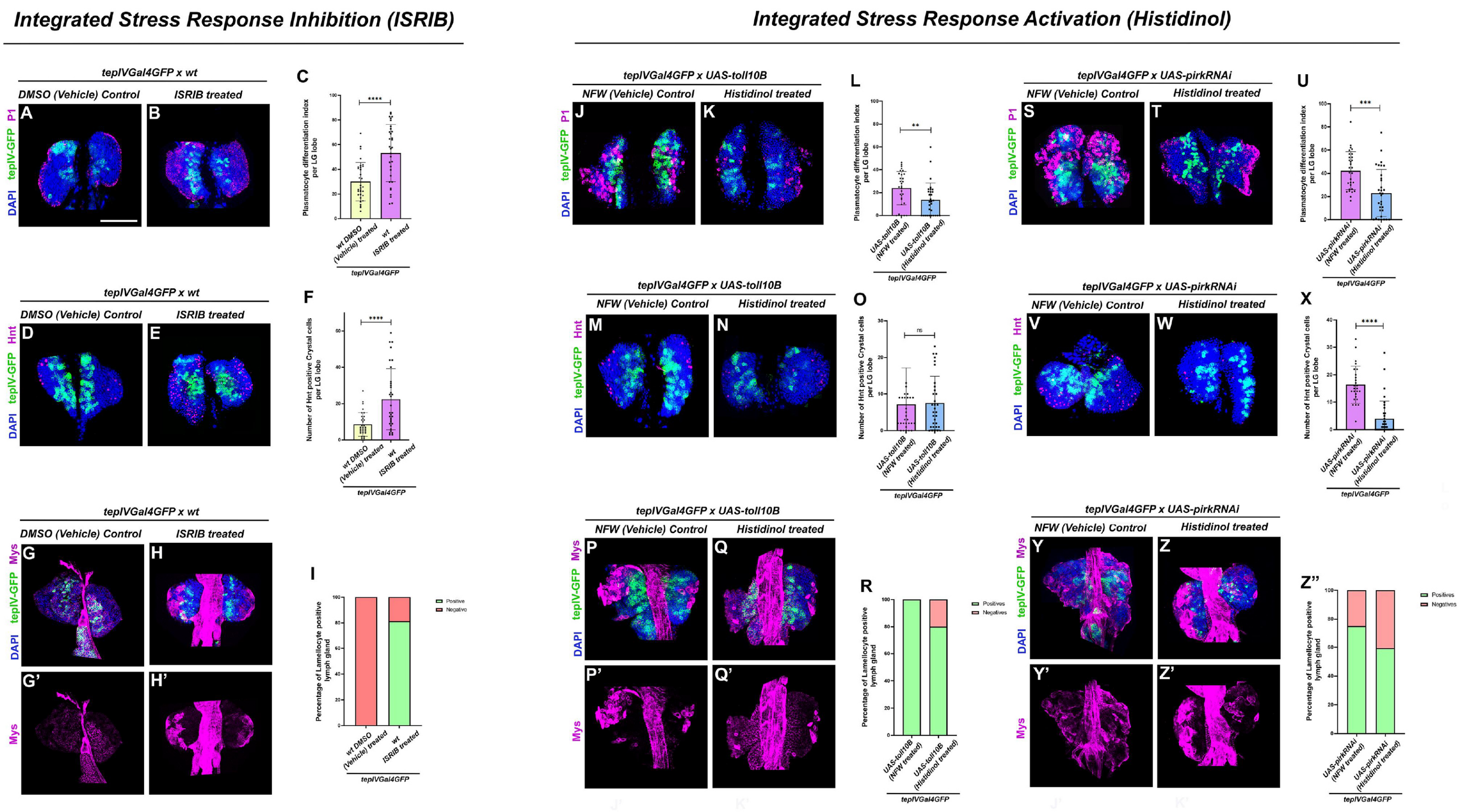

Fig. S4. Chemical modulation of ISR pathway regulates LG hematopoiesis

Plasmatocyte differentiation marked by P1 (magenta) or Crystal cell differentiation marked by Hindsight (Hnt in magenta) or lamellocyte differentiation marked by Myospheroid (Mys in magenta) in *tepIVGal4 x wt* larvae (Fig. S8B, E, H – H') upon ISR inhibition by ISRIB treatment as compared with DMSO (vehicle) control (Fig. S8A, D, G - G') or *tepIVGal4 x UAS-toll10B* (Fig. S8K, N, Q – Q') *or tepIVGal4 x UAS-pirkRNAi (*Fig. S8T, W, Z – Z'*)* larvae upon ISR activation by treatment with Histidinol as compared with respective NFW (vehicle) control (Fig. S8J, M, P – P', S, V, Y – Y'). Graphical representation of Plasmatocyte differentiation index (Fig. S8C, L, U) or number of Crystal cells (Fig. S8F, O, X) or percentage of Lamellocyte positive lymph glands (Fig. S8I, R, Z") in *tepIVGal4 x wt* larvae upon ISR inhibition by ISRIB treatment (Plasmatocytes: N = 16, n = 32; Crystal cells: N = 17, n = 33; Lamellocytes: N = 16) as compared with the respective vehicle control (Plasmatocytes: N = 16, n = 32; Crystal cells: N = 16, n = 32; Lamellocytes: N = 18) or *tepIVGal4 x UAS-toll10B* larvae upon ISR activation by Histidinol treatment (Plasmatocytes: N = 15, n = 30; Crystal cells: N = 16, n = 32; Lamellocytes: N = 17) as compared to the respective vehicle control (Plasmatocytes: N = 15, n = 30; Crystal cells: N = 15, n = 30; Lamellocytes: N = 16) *or tepIVGal4 x UAS-pirkRNAi* larvae upon ISR activation by Histidinol treatment (Plasmatocytes: N = 16, n = 32; Crystal cells: N = 18, n = 36; Lamellocytes: N = 32) were analyzed as compared to the respective vehicle control (Plasmatocytes: N = 15, n = 30; Crystal cells: N = 13, n = 26; Lamellocytes: N = 12). GFP (green) is driven by *tepIVGal4* which marks core progenitors in the LG (S8A-Z'). Nuclei are stained with DAPI (Blue). N denotes the number of larvae & n denotes the number of individual primary lymph gland lobes analysed per genotype. Individual data points in the graphs represent individual primary lobes of the Lymph Gland. Values are mean ± SD, and asterisks denote statistically significant differences (ns denotes not significant, **$p < 0.01$, *** $p < 0.001$, ****$p < 0.0001$). Student's t-test with Welch's correction was performed for the statistical analysis. Scale Bar: 50 µm (A-Z').

Biology Open • Supplementary information

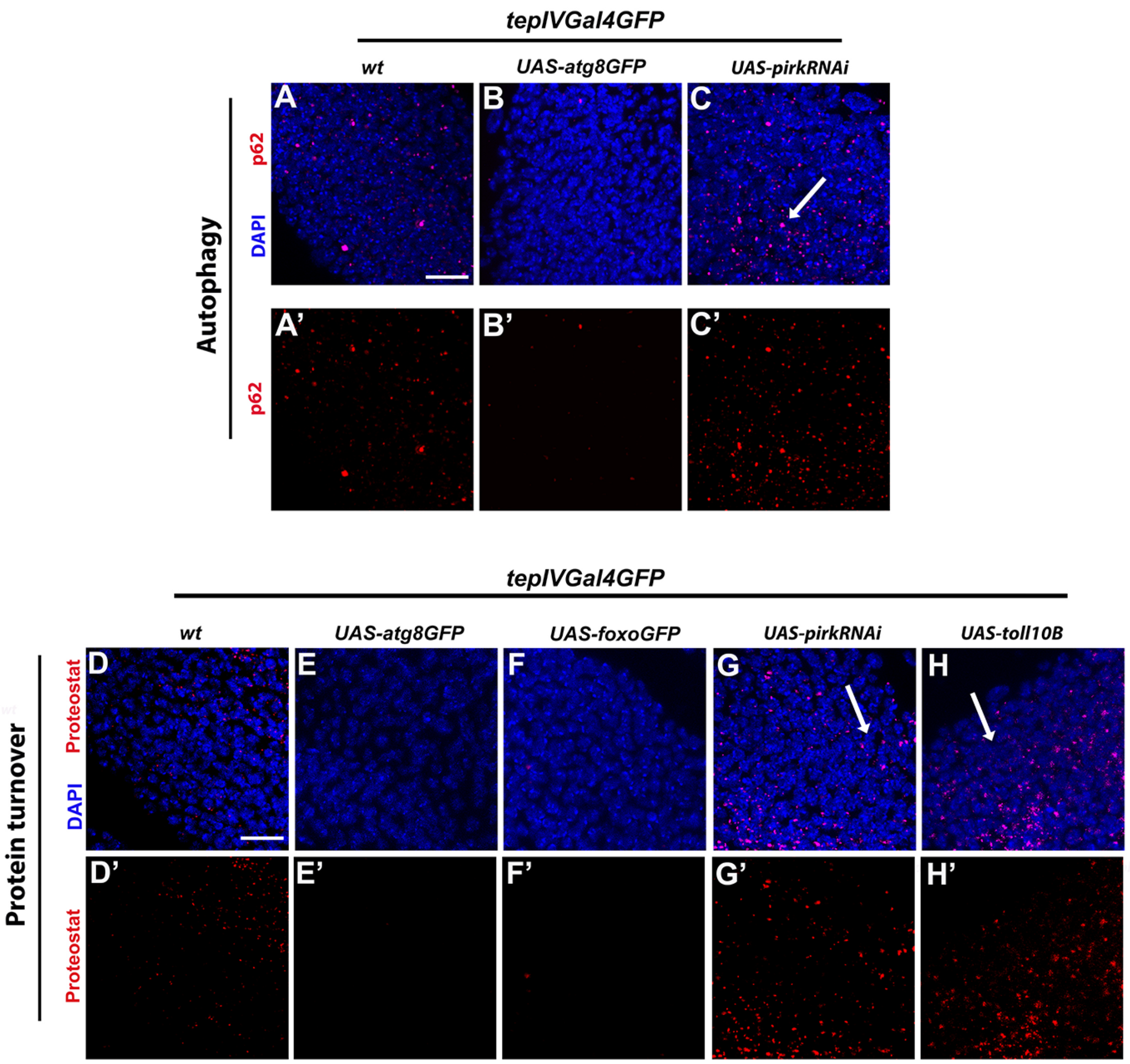

Fig. S5. Single channel images to visualize levels of autophagy and protein turnover upon modulation of cellular homeostasis in LG hematopoietic progenitors

Estimation of autophagy levels by evaluating the ratio of p62 positive punctae per cell marked by p62 antibody (red) upon core progenitor-specific (using *tepIVGal4*) knockdown of *pirk* or over-expression of atg8 as compared to wildtype control (Fig. 1A-C, A'-C'). Assessment of protein turnover by estimating the ratio of Proteostat positive punctae per cell marked by PROTEOSTAT detection reagent (red) upon *tepIVGal4* mediated expression of *UAS-atg8GFP*, *UAS-foxoGFP*, *UAS-pirkRNAi* or *UAS-toll10B* as compared to *wildtype* control (Fig. 1D-H, D'-H'). Nuclei are stained with DAPI (Blue, 1A-H). Scale Bar: 30 µm (A-C & A'-C', D-H & D'-H').

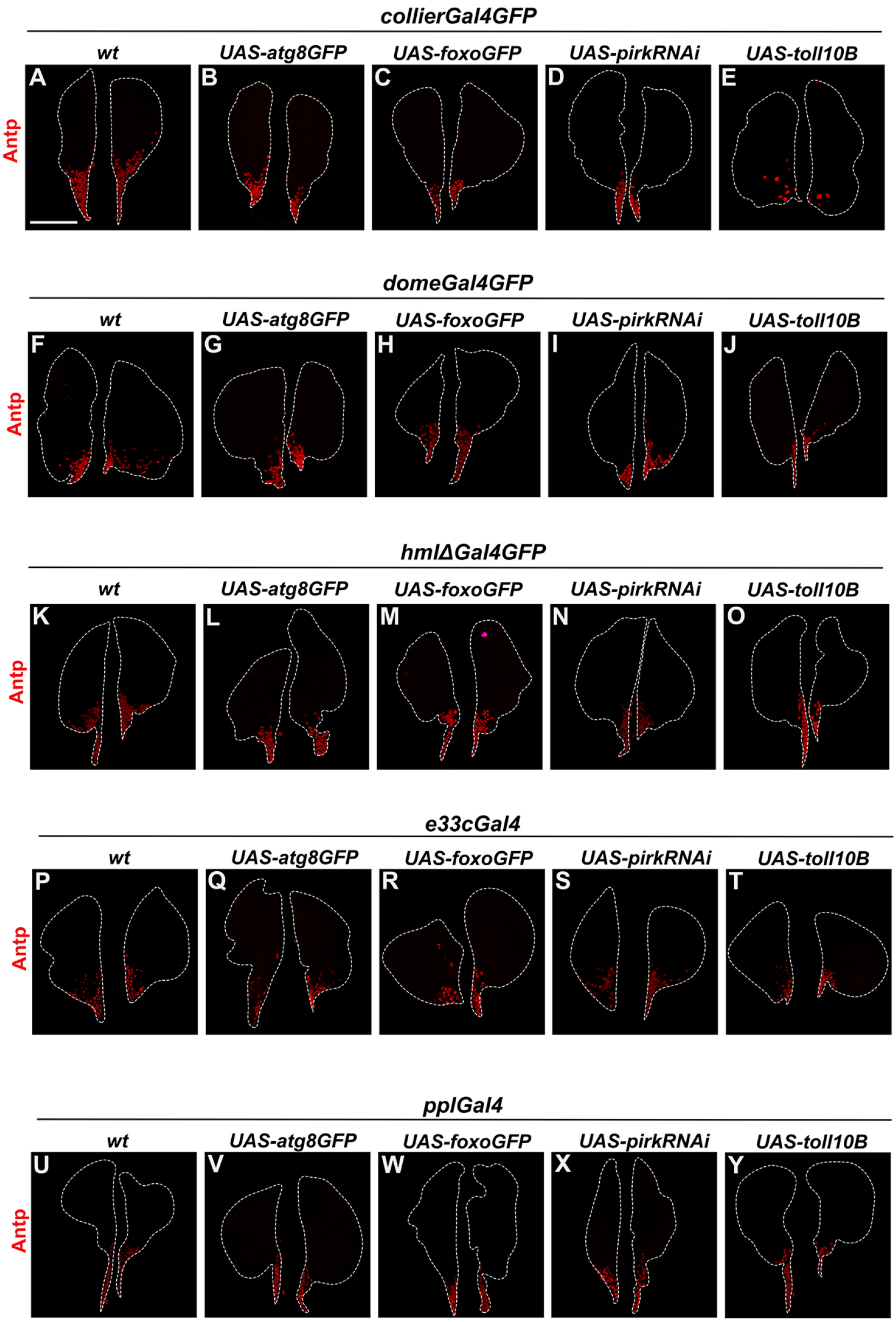

Biology Open • Supplementary information

Fig. S6. Single channel images showing the lymph gland PSC niche size upon localized perturbation or systemic modulation of cellular homeostasis

Posterior Signalling Centre (PSC) niche cell population marked by Antennapedia (red) upon either PSC- specific (using *collierGal4*) or hematopoietic progenitor- specific (using *domeGal4*) or cortical zone differentiated hemocytes- specific (using *hmlΔGal4*) or whole lymph- specific (using *e33cGal4*) or systemic fat body- specific (using *pplGal4*) expression of *UAS-atg8GFP* (2B-V), *UAS-foxoGFP* (2C-W), *UAS-pirkRNAi (2D-X)* or *UAS-toll10B* (2E-Y) as compared to respective *wildtype* control (2A-U). Scale Bar: 50 µm (A-Y).

Biology Open • Supplementary information
